# Letter to the Editor “Development and validation of a novel nomogram model for predicting postoperative survival of T4N0M0 NSCLC: a population-based survival analysis

**DOI:** 10.1097/JS9.0000000000003575

**Published:** 2025-10-06

**Authors:** Man Sun, Dan Zang, Jun Chen

**Affiliations:** Department of Oncology, The Second Hospital of Dalian Medical University, Shahekou District, Dalian, Liaoning, China

*Dear Editor,*

We read with interest the recent study by Lu et al^[1]^, which proposed a SEER-based nomogram to estimate postoperative overall and cancer-specific survival in patients with surgically resected T4N0M0 non-small cell lung cancer (NSCLC). As clinical interest grows in refining treatment strategies for resectable stage IIIA NSCLC, this study addresses an important and under-investigated subgroup. However, several methodological and translational limitations must be addressed before the model can be reliably integrated into clinical decision-making. This study complies with the TITAN 2025 guidelines for transparent and ethical reporting of AI-assisted research^[2]^.

First, the model’s binary classification of adjuvant therapies undermines its causal interpretability by failing to distinguish neoadjuvant versus adjuvant intent or specify regimens and radiation techniques. Such oversimplification masks clinically relevant heterogeneity. The reported association between postoperative radiotherapy and poorer CSS likely reflects confounding by indication – where higher-risk patients are more likely to receive intensive treatment – rather than a true adverse effect^[3]^. Similarly, including surgical type without adjusting for selection factors such as tumor location, comorbidity, or surgeon preference may distort outcome attribution^[4]^. Future models should incorporate granular treatment variables and apply methods such as propensity score adjustment or stratified modeling to mitigate selection bias and enhance clinical relevance.

Second, the exclusion of key molecular variables – such as EGFR, TP53, ALK, PD-L1, and postoperative ctDNA – critically limits the model’s relevance in the era of precision oncology^[5]^. Contemporary management of resected stage III NSCLC increasingly relies on molecular risk stratification and minimal residual disease assessment^[6]^. Trials such as IMpower010 and LUNGCA-1 have shown that ctDNA positivity outperforms TNM staging in predicting recurrence and informing adjuvant decisions. This molecular omission may also contribute to the model’s limited discriminative ability. Future models should integrate clinicogenomic data through SEER linkage with platforms such as TCGA, GENIE, or institutional registries.

Third, using Kaplan-Meier and Cox models to estimate CSS likely overstates lung cancer mortality by censoring non-cancer deaths – particularly in older patients with high competing risks such as cardiovascular or pulmonary disease. This may explain the observed divergence between CSS and overall survival in elderly subgroups. Incorporating competing risk methods, such as the Fine-Gray model, would yield more accurate and clinically interpretable prognostic estimates for surgically treated NSCLC^[7]^. This refinement is essential to ensure reliable risk prediction in real-world surgical cohorts.

Finally, the nomogram was not benchmarked against established staging systems such as the AJCC 8th edition TNM classification, leaving its incremental value uncertain. Despite reporting moderate discrimination (C-index: 0.712 for OS and 0.726 for CSS), the model’s performance was not directly compared with traditional frameworks using metrics like net reclassification improvement, integrated discrimination improvement, or decision curve analysis^[8]^. Without such validation, it remains unclear whether the proposed tool meaningfully enhances prognostic accuracy, risk stratification, or clinical decision-making beyond TNM staging. As claims of “individualized prediction” hinge on demonstrable added benefit, future studies should incorporate head-to-head comparisons to support clinical utility and inform surgical decision-making.

In summary, while Lu et al. advance efforts in individualized prognostication for surgically managed NSCLC, their model lacks key elements necessary for clinical adoption – namely analytic robustness, molecular granularity, and benchmarking against established frameworks. As thoracic oncology pivots toward precision surgery and biomarker-driven care, prognostic tools must evolve accordingly. This correspondence underscores critical limitations and advocates for models that are not only statistically rigorous and biologically informed but also readily embedded within real-world surgical workflows.

## Data Availability

No datasets were generated or analyzed for this article. Data sharing is not applicable.

## Research registration unique identifying number (UIN)

Not required.
